# Event-Based Trajectory Prediction Using Spiking Neural Networks

**DOI:** 10.3389/fncom.2021.658764

**Published:** 2021-05-24

**Authors:** Guillaume Debat, Tushar Chauhan, Benoit R. Cottereau, Timothée Masquelier, Michel Paindavoine, Robin Baures

**Affiliations:** ^1^CERCO UMR 5549, CNRS—Université Toulouse 3, Toulouse, France; ^2^Laboratory for Research on Learning and Development (LEAD), University of Burgundy, CNRS UMR, Dijon, France

**Keywords:** SNN, STDP, unsupervised learning, spiking camera, ball trajectory prediction, motion selectivity

## Abstract

In recent years, event-based sensors have been combined with spiking neural networks (SNNs) to create a new generation of bio-inspired artificial vision systems. These systems can process spatio-temporal data in real time, and are highly energy efficient. In this study, we used a new hybrid event-based camera in conjunction with a multi-layer spiking neural network trained with a spike-timing-dependent plasticity learning rule. We showed that neurons learn from repeated and correlated spatio-temporal patterns in an unsupervised way and become selective to motion features, such as direction and speed. This motion selectivity can then be used to predict ball trajectory by adding a simple read-out layer composed of polynomial regressions, and trained in a supervised manner. Hence, we show that a SNN receiving inputs from an event-based sensor can extract relevant spatio-temporal patterns to process and predict ball trajectories.

## Introduction

The original aim of Artificial Neural Networks (ANNs) was to mimic human or even non-human brain processing. The learning and generalization abilities of ANNs have led to great advances, particularly in solving visual tasks (Rawat and Wang, 2017). However, the quest for performance has taken ANNs away from their original bio-inspired function, even if ANNs show good performances with neural activity correlated with human cortical activity (Schrimpf et al., 2018).

There is, however, another category of neural networks, called Spiking Neural Networks (SNNs). SNNs use spikes as signals between neurons, and in this respect, are closer to the brain than ANNs. The temporality of these spikes provides additional information (VanRullen et al., 2005), making SNNs good candidates to deal with spatio-temporal stimuli. Moreover, since spiking activity is usually binary-coded and sparse (Van Rullen and Thorpe, 2001; Perrinet et al., 2004), processing in SNNs is highly power efficient (Rueckauer et al., 2017; Barrios-Avilés et al., 2018; Pfeiffer and Pfeil, 2018).

SNNs can be coupled with synaptic plasticity rules such as STDP (Spike Timing Dependent Plasticity), which are bio-inspired and unsupervised.

SNNs with STDP rule have been applied many times on image categorization tasks, in order to benchmark them against more common ANNs or other SNNs (Diehl and Cook, 2015; Kheradpisheh et al., 2018; Lee et al., 2018; Thiele et al., 2018). However, most of these studies used static images as stimuli, and thus, did not take full advantage of the above-mentioned benefits. In contrast, videos (or spikes from spiking cameras also called event-based cameras) are more suited for SNNs due to their spatio-temporal nature (Pfeiffer and Pfeil, 2018; Iyer et al., 2021). Recently, (Orchard et al., 2017) used event-based cameras and mimicked retinal saccades to perform categorization tasks on standard datasets like MNIST. This allowed spike processing on more biologically plausible and more realistic data where a temporal dimension was induced directly by the motion saccade. The performance of the model was not only excellent (Lee et al., 2016), but even surpassed state-of-the-art ANNs on temporally occluded images (Moraitis et al., 2020).

In order to process motion, frame-based cameras are the most common way to acquire data. Frame-based processing is different from that of the retina. Typically, the camera output is synchronous, and processed by the ANN frame-by-frame. This processing then induces response time delays depending on the number of frames per second (FPS), motion blur, and data redundancy, resulting here again in unnecessary resource consumption.

However, the development of visual (Lichtsteiner et al., 2008; Posch et al., 2011; Brandli et al., 2014; Son et al., 2017), audio (Liu et al., 2014), and tactile (Taunyazov et al., 2020) event-based sensors brings them closer to biomimetics. These sensors only encode variations (of brightness, frequency, etc.) and are fully asynchronous, much like the retina. This allows sensors to generate extremely sparse data and to considerably reduce response latency (Farabet et al., 2012). These sensors make it possible to take advantage of all the benefits of SNNs. Indeed, a combination of SNNs with event cameras has been used to solve several tasks, such as object detection (Bichler et al., 2012), optical flow estimation (Orchard et al., 2013; Adams and Harris, 2014; Paredes-Valles et al., 2019), motion detection, etc.

These studies show that a bio-inspired system composed of an SNN driven by inputs from an event-based camera can learn, in an unsupervised manner, to optimally process spatio-temporal data.

In this study, we used a specific type of event camera, the “Neurosoc,” introduced in section Choice of the Event Camera. We recorded ball trajectories with the NeuroSoc and used an SNN to learn specific features of the trajectory (direction, speed, shape). Our objective was to test the accuracy of this setup in predicting the arrival point of the ball under various presentation times. We wanted to test if our network is able to anticipate the arrival point of the ball based on a snapshot of the trajectory, like sport experts do on the field, for example (Farrow and Abernethy, 2003).

A ballistic trajectory is constrained by physical laws, and based on these regularities. Humans can anticipate the arrival point of a moving object from information about the object's position, velocity and direction (Aglioti et al., 2008). Likewise, in this study, we aimed to decode the output of the SNN with polynomial regression. If after learning, neurons code for precise directions and speeds, it should be possible to accurately predict where the ball will fall from the SNN responses.

## Materials and Methods

**Figure 1:**
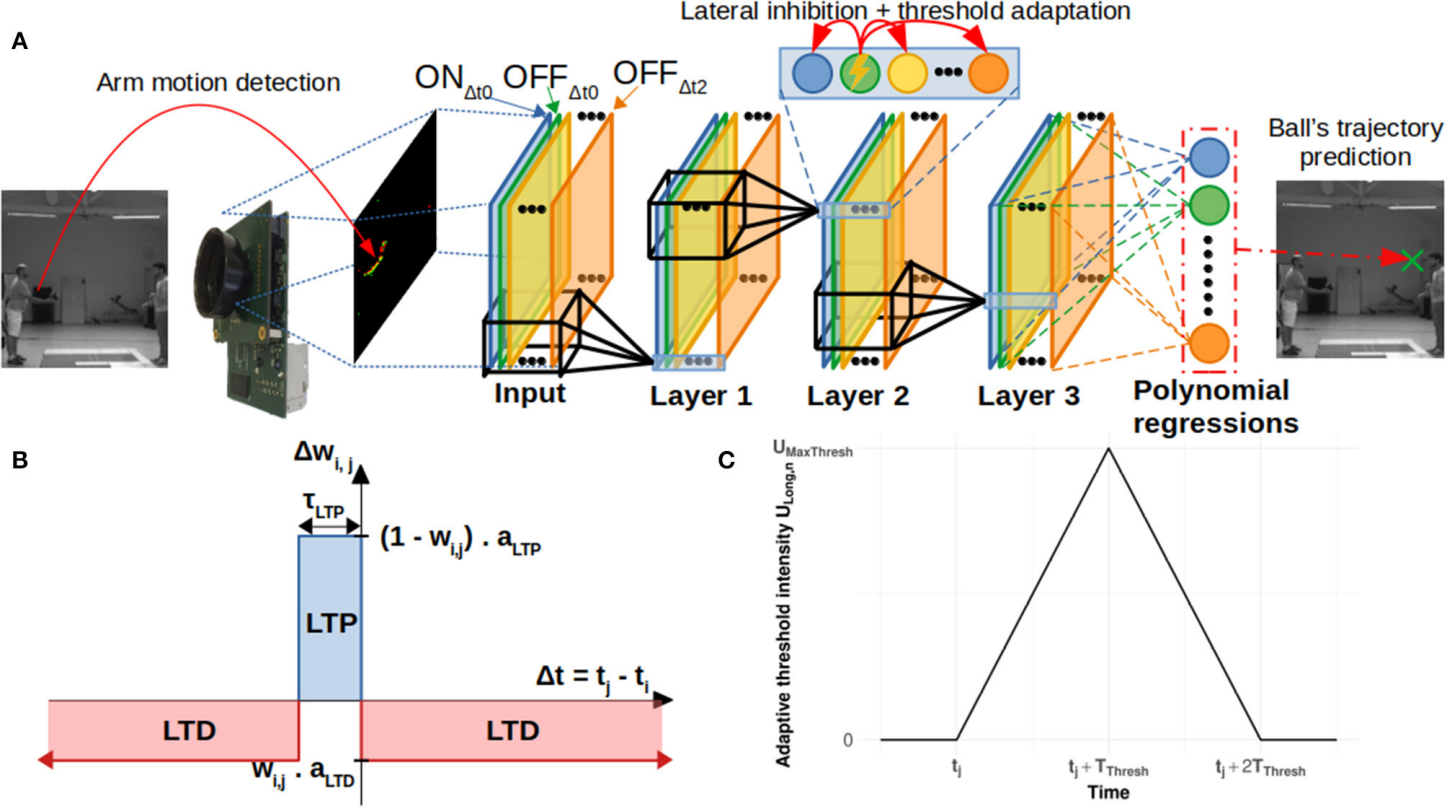
**(A)** General architecture of the system. The neurosoc camera captures “ON” and “OFF” events (red and green points). These are duplicated, delayed (three different delays are used: Δt0, Δt1, and Δt2) in the input layer (see section Delays) and subsequently sent to a 3-layers SNN. Output spikes are finally processed by a read-out layer in order to predict the final position of the ball. **(B)** Simplified STDP learning rule. **(C)** Evolution of the adaptive threshold intensity U_Long, n_ over time (see section Lateral Inhibition and Threshold Adaptation).

The aim of this study was to predict the ending point of a ball's trajectory from an artificial visual system. Our pipeline consisted of (1) a “Neurosoc” camera which generates spikes from ball trajectories, (2) a 3-layer SNN equipped with an STDP learning rule which progressively becomes selective to motion patterns and (3) a read-out layer which uses polynomial regressions to recover the ending point of the ball's trajectory. We further detail these three parts in the next sections. Figure 1 provides a schematic overview of the artificial system.

### Neurosoc

#### Choice of the Event Camera

Several models of event-driven cameras have already been proposed in the industry (Prophesee, iniVation, Insightness, Samsung, CelePixel) and operate mainly according to a pixel-to-pixel temporal difference. Although the performances of these devices are remarkable in terms of temporal frequency and dynamic range, they suffer from some crucial limitations in terms of bio-inspired modeling, namely the inexistence of spatial filters upstream of the spike-generation. Using bio-inspired models which capture various aspects of the visual system (Masquelier and Thorpe, 2007), these filters make it possible to reinforce the performances of spike-based analysis by introducing a bio-inspired component upstream of the spike-generation.

In parallel to this observation, different devices have appeared during the last few years which allow the generation of spikes from standard CMOS image sensors (Abderrahmane and Miramond, 2019; Admin, 2020; Spike Event Sensor, 2021). The main objective behind these cameras is, on one hand, to be able to integrate spatial filters upstream of the spikes generation, and on the other hand, to have sensors of different formats going, for example, up to 2M pixels (Caiman Camera, 2021) [usually the pixel-count of event-based cameras is rather limited (Lichtsteiner et al., 2008; Posch et al., 2011; Brandli et al., 2014; Son et al., 2017)].

In order to guarantee reliable integration of spikes, it is imperative that these image sensors work in global-shutter mode (instantaneous image acquisition), and with a short exposure time (in the order of few milliseconds). Such cameras are an intermediary between event-based sensors and frame-based cameras, and allow for spatial and temporal filtering with high FPS.

The NeuroSoc camera from Yumain (Spike Event Sensor, 2021) possesses these characteristics and was, therefore, chosen for this study. This camera operates at 240 frames per second at a resolution of 128 × 120 px. Spatial and temporal filters are embedded in a processing board closed to the image sensor (see section Architecture of the NeuroSoc Event Camera) to detect brightness variations and generate spikes. As explained above, these spatial filters (here DoG type) are closer to those found in the lateral geniculate nucleus in the human visual system. As a result, they reduce noise, detect edges, and increase output sparseness.

#### Architecture of the NeuroSoc Event Camera

**Figure 2:**
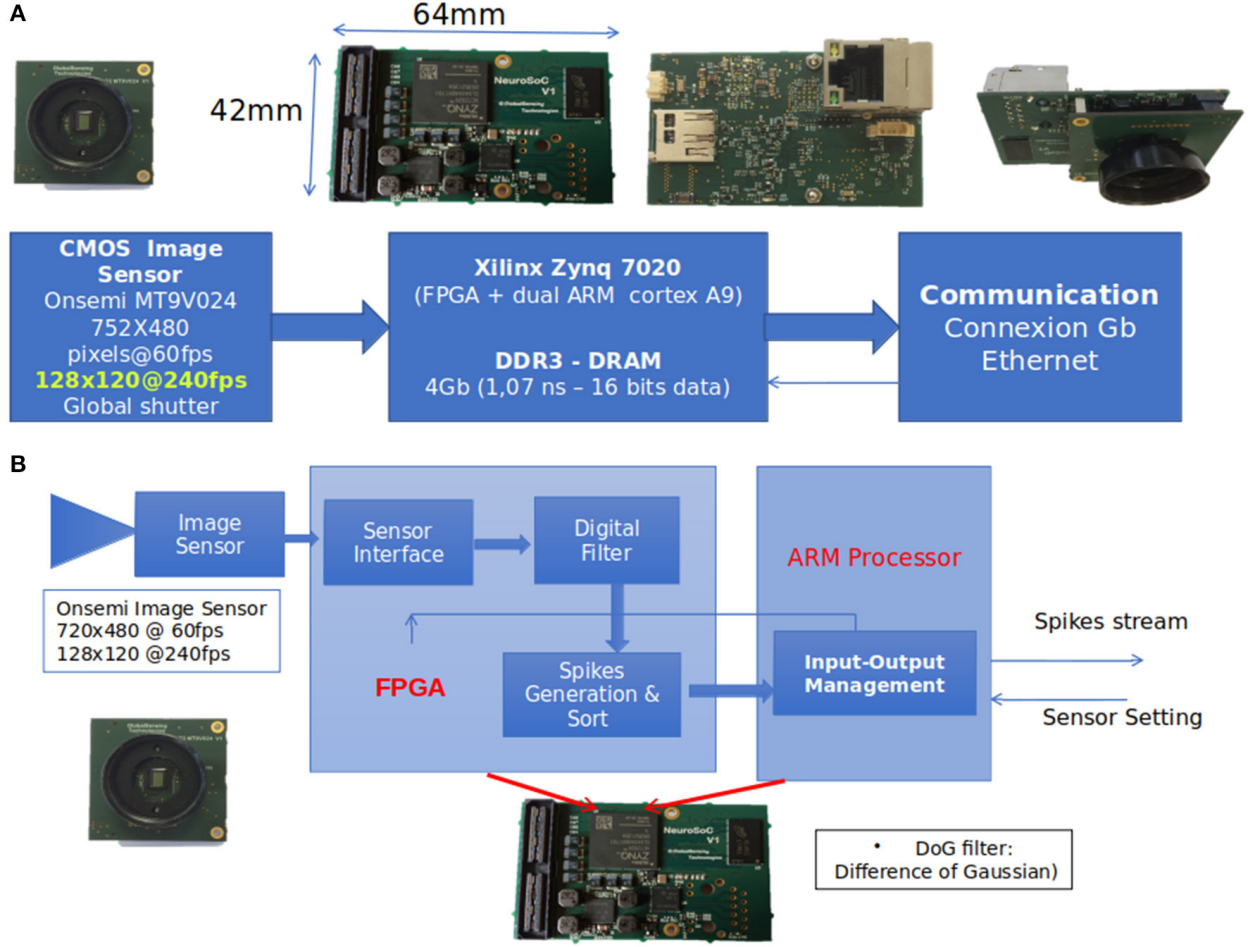
**(A)** The event-based camera used in the study (NeuroSoc). It is based on a global shutter CMOS image sensor connected to a MPSoC Zynq circuit. **(B)** Implementation treatments on NeuroSoC board.

We used the NeuroSoc event camera developed by Yumain which is based on a global-shutter CMOS MT9024 image sensor from On Semiconductor and a board called NeuroSoC. This board is composed of a MPSoC Zynq 7020 circuit from Xilinx and a 4 Gbits DDRAM memory (see Figure 2). The CMOS sensor operates in global-shutter mode (instantaneous image acquisition) with an exposure time in the range of 31 ns to 4 ms. In the context of this study, images are generated in a 128 × 120 pixels format guaranteeing a throughput of 240 frames per second, with an exposure time of 3.7 ms due to low luminosity conditions (window shutters closed for proper operation of the Vicon). Images from the image sensor transmitted to the Zynq MPSoC circuit are filtered in real time in order to extract the salient parts of the objects contained in the images. The first step of the process consisted of calculating the difference between the images at time t_n_ and t_n−1_ (the sampling period t_n_-t_n−1_ was 4.17 ms or 1/240 fps). In this study, a DoG (Difference of Gaussian) filter was applied to this difference. The output of the filter was classified (positive/negative values generate ON/OFF spikes) and sorted according to the most important to the least important absolute values above a threshold, thus constituting a train of temporal spikes. The threshold value was set manually during the acquisition phase, and was adjusted to extract as many spikes from movements as possible while keeping the noise level low. As shown in Figure 2, all these treatments are implemented in the FPGA within the Zynq MPSoC.

The spike stream was transmitted outside the camera *via* an ethernet link. Input/output management (spike transmission, sensor exposure time control) of the event camera was performed through the ARM processor of the Zynq MPSoC.

#### Time Encoding

The outputs of the filters implemented in the cameras (see above) were first thresholded. Values above threshold were subsequently converted into spikes using an intensity to latency conversion (Thorpe et al., 2001; VanRullen et al., 2005; Masquelier and Thorpe, 2007; Chauhan et al., 2018). Spike-latencies were obtained by inverting the output values of the corresponding filters. Spikes were spread between the current and the next frame, see Equation (1):

(1) $$\begin{array}{l} \Delta t_{s,rel}=\frac{\left(I_{s}-I_{min}\right)}{FPS\cdot \left(I_{max}-I_{min}\right)}\text{and }t_{s}=\frac{F}{FPS}+\Delta t_{s,rel} \end{array}$$

With:

F = Index of the current frame
Δt_s, rel_ = Relative time of the spike *s* to the current frame *F*
I_s_ = Inverted intensity of the spike *s*
t_s_ = Time of the spike *s*.

### SNN

In this study, the architecture we used is similar to other proposed multi-layer convolutional SNN (Masquelier and Thorpe, 2007; Tavanaei and Maida, 2017; Kheradpisheh et al., 2018; Mozafari et al., 2018; Thiele et al., 2018; Paredes-Valles et al., 2019). These studies highlighted the relevance of multi-layer SNN trained with an STDP learning rule to extract spatiotemporal features. Our architecture is composed of 3 layers trained with STDP. There is no pooling layer, and the network uses delays similar to (Paredes-Valles et al., 2019), but directly applied to the input layer generated by the NeuroSoc (see Figure 1A). We used a 3-layer SNN, composed of leaky integrate and fire (LIF) neurons with feedforward connections and lateral inhibition. The synaptic weights of the feedforward connections were learnt through a simplified STDP rule (Bichler et al., 2012). Similar as (Masquelier and Thorpe, 2007; Paredes-Valles et al., 2019), we used a weight sharing process with retinotopically organized neurons connected to 5 × 5 × d patches and a stride of 1 (d corresponds to the number of convolutional filters in the previous layer). The network was also endowed with a lateral inhibition mechanism which reduced the membrane potential of all neurons sharing the same position, instead of resetting it (see details in section Lateral Inhibition and Threshold Adaptation). Simulations were performed using a C++ code developed by the team.

#### Neuron Model

Our SNN was based on leaky integrate and fire (LIF) neurons. When such a neuron receives an incoming spike, its membrane potential increases in proportion to the synaptic weight that connects it to the pre-synaptic neuron that emitted the spike. In the absence of incoming spikes, the neuron membrane potential leaks according to Equation (2):

(2) $$\begin{array}{l} U_{i}\left(t\right)=U_{rest}+\left(U\left(t_{k}\right)-U_{Rest}\right)\cdot exp\left(\frac{-\left(t-t_{k}\right)}{\tau_{memb}}\right) \end{array}$$

t_k_: last time update
U_Rest_: resting potential = 0

The SNN was event-based, and the membrane potential of a neuron was only updated when an incoming spike was received by the neuron. First, the leak was applied, and then, a value W_i, j_ (weight connection between neuron i and j, constrained between 0 and 1) multiplied by W_Max_ was added to the membrane potential.

A given neuron emitted a spike when its membrane potential reached a threshold value *U*_*Thresh*_. A spike was then generated and propagated to the next layer and the membrane potential was reset to its resting-state value *U*_*rest*_.

#### STDP

To update synaptic weight connections and to give neurons the ability to learn specific features, we used a bio-inspired unsupervised learning rule called spike-timing dependent plasticity (STDP) (Bi and Poo, 2001; Caporale and Dan, 2008). It detects input correlations and enables the neurons to become selective to most frequently occuring patterns. Previous studies have already demonstrated that SNNs equipped with STDP can learn repetitive specific patterns (Masquelier et al., 2008), visual properties such as orientation (Delorme et al., 2001; Masquelier, 2012), binocular disparity (Chauhan et al., 2018) or shape (Masquelier and Thorpe, 2007; Diehl and Cook, 2015; Thiele et al., 2018). In this study, we used a simplified STDP learning rule inspired from (Bichler et al., 2012) (see Figure 1B). This simplified version does not include a time window for LTD. If the time of the last presynaptic spike is not included within the LTP window (τ_LTP_), LTD is applied. Synaptic weight updates corresponding to spikes occurring during the LTP window are all equal and therefore independent of spike times, as shown in Equation (3). Contrary to the synaptic update rule used in (Bichler et al., 2012), we included a multiplicative term which depends on the current weight. This multiplicative rule also forces a soft-bound on the weights in the interval (0, 1).

(3) $$\begin{array}{l} \Delta W_{i,j}=\left(1-W_{i,j}\right)*a_{LTP}\;if\;t_{j}-t_{i}<\tau_{LTP}\text{ and}\\ \Delta W_{i,j}=-W_{i,j}*a_{LTD}\;otherwise \end{array}$$

W_i, j_: weight synaptic connection between afferent neuron i and j
t_i_: last spike of afferent i.
t_j_: last spike of neuron j.
a_LTP_/a_LTD_: amplitude of the potentiation/depression
τ_LTP_: LTP time window.

#### Delays

Although SNNs equipped with STDP can learn different spatial patterns (orientation, spatial frequency, binocular disparity, …), learning motion direction is a harder task because it relies on spatio-temporal properties. Indeed, input neurons with similar spatial positions spike but not in the same order: for leftward (or rightward) motion, inputs placed on the left (right) spike first, followed by other inputs from the left (right) to the right (left). The common way to improve SNNs' temporal selectivity and thereby permit discrimination of motion direction is to use delays. These delays improve synchrony between input spikes for specific spatio-temporal patterns (rightward motion, for example) and desynchronize inputs for opposite patterns (leftward motion).

In order to work with delays, it is necessary to be careful about the strategy employed to learn them. Over the last few years, various approaches have been proposed to select these delays (Eurich et al., 2000). For instance, the *delay shift* (Eurich et al., 2000; Tversky and Miikkulainen, 2002; Gibson et al., 2014) approach consists of learning the delay from the input spikes. In addition to the weight-learning rule, this approach also uses a specific learning rule for delays to increase the simultaneity of the input spikes. Another approach, called *delay selection* (Paredes-Valles et al., 2019), consists of duplicating synapses and adding delays to them. In delay selection, a single weight-learning rule can be used to select synapses with appropriate delays. In this study, we used this second method (Eurich et al., 2000; Paredes-Valles et al., 2019). Three different delays (0, d1, and d2) were applied on each input and each synapse was therefore duplicated three times. Because we consider both “on” and “off” events, there were 6 different synapses for each pixel-positions (ON_Δ*t* = 0_, OF*F*_Δ*t* = 0_, ON_Δ*t* = *d*1_, …, OF*F*_Δ*t* = *d*2_) as shown in Figure 1A.

#### Lateral Inhibition and Threshold Adaptation

When a neuron spiked, the neurons of the same layer sharing the same retinotopic position were prevented from spiking. We used an instantaneous inhibition, the membrane potential was reduced by a specific value U_Inst_ [see, e.g., Diehl and Cook (2015) and Delorme et al. (2001)]. The purpose of this inhibition was to prevent the filters from learning similar patterns. It therefore increases selectivity and sparsity within the neural population.

We also used a homeostatic process which added a penalty U_long, n_ to the neurons by increasing their threshold. The magnitude of change in the threshold had an inverted “V” shape in time—it increased and subsequently decayed back to zero (the increase and decrease both lasted for a time-interval T_Thresh_), allowing a time dependent threshold adaptation (see Figure 1C and Annex 1 in Supplementary Material).

The threshold variation and inhibition intensity was made proportional to U_PropInh_: the current average of squared membrane potentials of neurons connected to the same patch (see Annex 1 in Supplementary Material).

#### Training Procedure

The 3-layer SNN was trained layer-by-layer in an unsupervised manner using the STDP learning rule described in section Delays. Seventy percentage of the recorded trajectories were used for training. During the training and testing phases, trajectories were presented individually and were separated by periods of 2 s without spikes, allowing the SNN to reset to its initial state. The third layer output was then used to make predictions, as detailed in section Trajectory Prediction. For each convolutional filter (N_f_ = 100 for the third layer), a polynomial regression was performed with the spatial position (x and y) of the spiking neuron as input and the ball's vertical position as the predicted variable.

An SNN with STDP learning rule has some parameters which need to be tuned (membrane potential time constant, ratio LTP/LTD, learning rate, …). We estimated these parameters with a genetic algorithm (Mohammadi et al., 2017) (see Table 1). A simplified version of the prediction process presented in section Trajectory Prediction was used. The cost function for the minimization was the average value of the mean prediction error at 15, 45, …, 90% of trajectory presentation.

**Table 1:** Parameters for each SNN's layers.

	**w_MAX_**	**τ_MEMB_**	**N_F_**	**τ_LTP_**	**a_LTP_**	**a_LTD_**	**f_INST_**	**f_LONG_**	**T_THRESH_**
Layer 1	1.873	0.01	60	0.0754	0.00195	0.0005	3.0	2.89	0.031
Layer 2	0.813	0.052	80	0.0236	0.0131	0.00118	2.82	2.41	0.023
Layer 3	1.308	0.039	100	0.0368	0.00815	0.00198	3.46	1.9	0.051

### Data Acquisition and Comparison

We recorded 297 passes of the ball between two participants using the Neurosoc camera. The two participants were separated by a distance of ~2.30 m, and the trajectories included multiple velocities. The camera's direction was perpendicular to the trajectories.

Each of the 297 trajectories was manually labeled so as to determine the start and end points of the throw, the direction and the height of the reception (X and Y-axis, respectively, see section Trajectory Prediction). The trajectories were partitioned using a 70–30 train-test split, and the final training and testing sets contained 208 and 89 trajectories, respectively.

To quantify the neuron's selectivity, we recorded the ball's 3D spatial position with Vicon cameras (Merriaux et al., 2017). These cameras use infrared light to capture the position of markers placed on the ball, at a frequency of 200 Hz. Four targets were placed at four opposite positions on the ball. The ball's center of gravity was obtained by averaging these positions. A low-pass filter was applied to these values with a cut-off frequency of 6 Hz. Using the exact value of the ball over time, we could easily determine the ball's direction and speed.

Vicon technology uses infrared flashing lights to detect targets. These lights were perceived by the camera and generated big variations of brightness which generated spikes. To overcome this issue, we used an anti-IR lens with a cut-off wavelength of 730 nm (SP730 Near-IR/Colorless Dichroic Block Shortpass Filter, 2015).

### Trajectory Prediction

Our main objective was to predict the ball's reception point from the output of the SNN. Since this point would be highly impacted by the receiver (depending on whether he moved his arms forward to intercept the ball or not), we restricted these predictions to the y-axis. The prediction along the x-axis was simplified, with only two choices reflecting the ball's direction (right or left).

**Figure 3:**
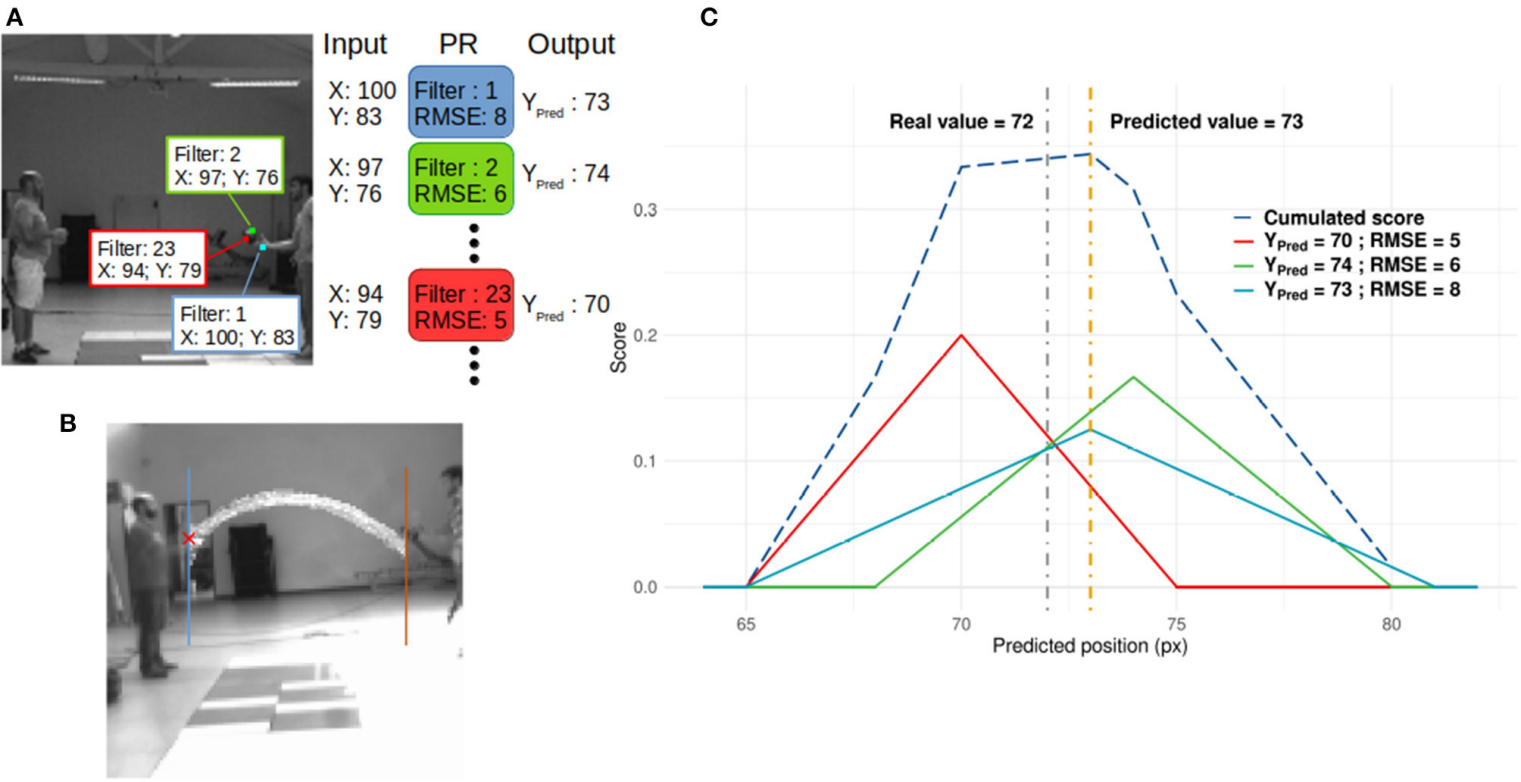
**(A)** Readout process. When a neuron spikes, its spatial position in its corresponding neural map is used as input for the PR linked to the spiking neuron's filter. In this figure, 3 neurons from different positions and filters spike and generate 3 predictions. These are then integrated to compute a score value as presented in **(C)**. **(B)** Horizontal positions used as receiving points. **(C)** Example of the score update (see Annex 2 in Supplementary Material). Thick lines represent the score added by three spiking neurons from different filters, based on Equation (4). It is assumed that these three neurons spike at the same time, and there is no leak of the score. The dashed blue line represents the score. The gray and orange dotted lines are the real and predicted values, respectively.

The y-value was the ball's position when it crosses the blue/brown line for leftward/rightward directions (see Figure 3B). Polynomial regressions (PR) for each filter of the last layer were used to decode the SNN output and predict the y-value. We used a simple decoder to ensure that performances were mostly driven by processing within the SNN. More complicated decoders could provide better results, but this study focuses on the performance of the SNN. Second degree polynomial regressions were chosen because filters of the SNN spiked for specific patterns. If the SNN did not develop any motion direction selectivity, predictions from PRs would be inaccurate. To demonstrate this point, we also applied PRs to the outputs of the neurosoc camera (i.e., PRs were performed on the SNN inputs) (see **Figure 6**).

In this study, to determine the prediction on the Y-axis, we used a PR of second degree for each filter *n*, Equation (4):

(4) $$\begin{array}{l} Y_{Pred,n}=a_{00}+a_{10}x+a_{01}y+a_{20}x^{2}+a_{02}y^{2}+a_{11}xy \end{array}$$

With x and y, the spatial position of the spiking neuron of filter n, as schematized in Figure 3A. PR's parameters were learned by presenting all spatial positions of spiking neurons of corresponding filter n as PR's input and the corresponding y-value at the spike time as value to predict. This process was applied for each filter of the output layer.

The ball's predicted direction (X_pred_) was not taken into account during the PR. Filters mostly encode for specific directions. The direction was thus independent of the PR. The root mean square error (RMSE) of each PR was then computed in order to evaluate the reliability of each PR. Finally, a scoring mechanism (see Figure 3C and Annex 2 in Supplementary Material) was used to spatially integrate the predicted value Y_Pred, n_ based on the PR's reliability, and perform an average prediction over time.

### Unsupervised Motion Tracking

The motion selectivity of filters should allow us to track specific motions of stimuli based on their speed, directions, shape, etc. There are few different motions-patterns in the stimuli, mainly the ball and arms which can be divided into multiple parts (hands, forearm, …). An ideal way to evaluate our network's ability to track specific motions should be to label all of them, but this would make the task highly time-consuming and unfeasible. This study mainly evaluates if filters spike for the ball or for shapes similar to the ball.

A first way to determine whether a filter spikes for the ball is to compute the distance between the center of the ball and the spike's position. Because the camera was facing the plan of the ball trajectory, there is a linear relation between the ball's position in the reference frame of the camera and the one of the Vicon. So, given the ball's position in the reference frame of the Vicon, we can easily compute its position on the frame and compute the distance between a given spike and the center of the ball.

Then, by looking at the mean distance D_n_ from the ball of all spikes for each filter n, we can determine if the given spike encodes the ball's motion (if D_n_ is very low) or some other feature like a part of an arm.

Because a neuron is determined by its position and its filter, which encodes or not for ball motion (based on D_n_), we should be able to track the ball's motion based on the output of this network. As an example, in the context of Figure 3A, we can expect that filter 2 and 23 could be used to track ball motion and filter 1 for hand motion.

### Comparison With Human Performance

In order to compare the performances of our system to human capabilities, we conducted an experiment during which 12 participants (mean age = 27 +/− 12,9) were instructed to predict the end-point of the ball at different time steps. The NeuroSoc camera also recorded the classical frame-based video at f = 240 fps in parallel with spike estimation and transmission. The trajectories presented to these participants were therefore exactly the same as the ones used to test our SNN. All participants had normal or corrected-to-normal vision, and were healthy and without any known oculomotor abnormalities. Participants were naïve with respect to the purpose of the experiment, which received the appropriate ethical authorization from the “Comité d'éthique de la Recherche” of the Federal University of Toulouse (agreement 2020-279). The sample size was determined using G^*^Power (Faul et al., 2007) after having analyzed the results of a previous experiment investigating the influence of presentation duration on anticipation's performances. The results showed that for a desired power of 0.90, a total sample size of 12 participants was required.

Only a part of the trajectories (i.e., the first 15, 30, 45, 60, 75, and 90%) were presented to the participants who were instructed to predict the end-point by clicking with the mouse on the anticipated ending point, without any temporal constraint. The experiment was divided into three parts:

- Pre-learning: first time videos are presented to participants
- Active-learning: after each prediction made by the participant, the exact arrival point was -shown on the screen to provide the prediction error to the user, as a feedback
- Post-learning: same procedure as Pre-learning (no error was shown) but subjects had the experience of the “Active-learning” phase.

For each part, 8 trajectories were shown for each percentage of presentation time, for a total of 48 trials in each condition. Videos with a dimension of 384 × 360 pixels were presented on a 13.3-in. screen (60 Hz, full resolution 1,366 × 768, dimension 29.5 × 17 cm in horizontal by vertical).

In contrast to our SNN, the human participants already had experience with ball motion or motion in general. We nonetheless included an active-learning phase in the experiment so that participants could adapt to its specificities.

## Results

### Selectivity

During the acquisition of the videos, we used the Vicon technology to precisely measure the trajectory and position of the ball (see section Data Acquisition and Comparison). After the learning phase, we characterized the selectivity of the neurons from these ground truth data. We averaged all results over 6 simulations of the SNN with different random weight-initializations. The network learned using the training set, and the results below are the analysis from the output of the third layer with the test set as input of the SNN.

#### Direction Selectivity

**Figure 4:**
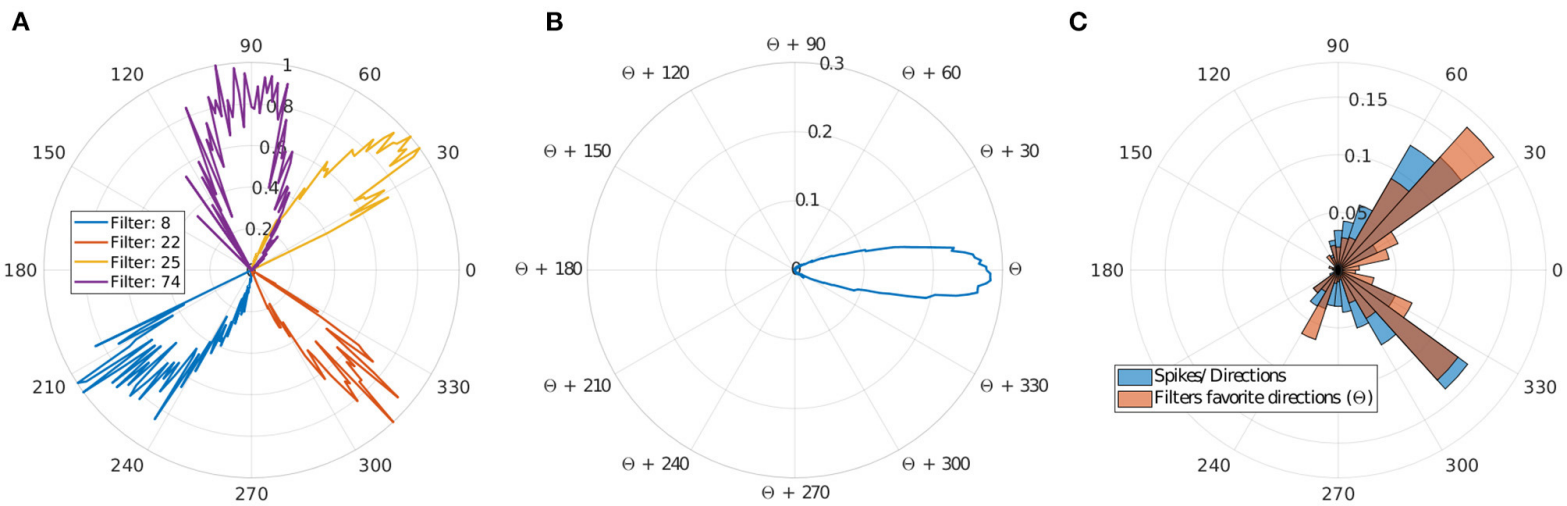
Direction selectivity. **(A)** Normalized direction selectivity θ*F*_n_ of four different filters. Each filter spikes for different direction's range. **(B)** General DS of all filters centered on their preferred direction θc_n_. **(C)** Distribution of the preferred directions of the filter compared to number of input spikes per direction.

Motion direction selectivity was obtained by counting the number of spikes from the output layer triggered by all the ball's trajectories. For each filter n, we divided the number of spikes for each direction of the ball (rounded to the unit) by the number of occurrences of each of these directions during all presented throws, giving us θ*f*_n_. These results are presented in Figures 4A,B.

We can observe that filters mostly spike for directions similar to their preferred direction θc_n_, which demonstrates a strong motion direction selectivity. Figure 4C, provides a comparison between a histogram of the preferred directions θc_n_ of our filters and the occurrence of input spikes for each direction.

Different filters are selective to different trajectories as shown in Figures 4A,C, and there are more filters selective to ascending motion. Indeed, leftward/rightward rising directions (which include the throw phase, when the ball is still in the thrower's hand) represent the majority of our trajectories, and this result confirms the ability of our model to learn the direction selectivity patterns in the inputs.

#### Speed Selectivity

**Figure 5:**
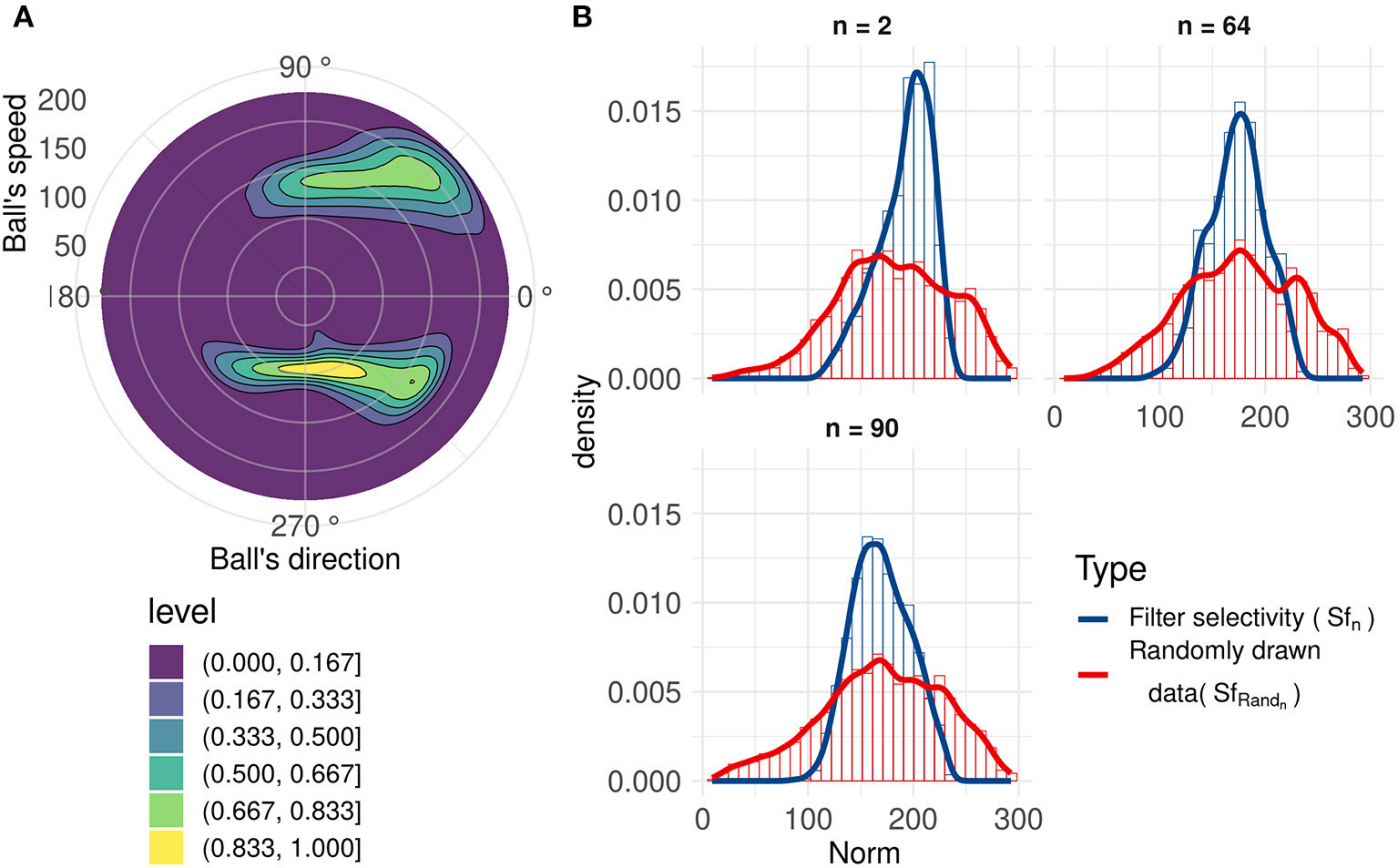
**(A)** 2D normalized density of the ball's speed by angle. Speeds and directions are correlated: for example, ascending motions are on average faster (initiation of the throw, angles around 45 and 315 degrees) than horizontal motion (top of the trajectory, angles around 90 and 270 degrees). **(B)** Range of speeds that generate a spike (blue line) for three filters with similar motion direction selectivity, compared to the range of speeds when the ball's direction causes the corresponding neuron to generate a spike (red line). These filters have similar direction selectivities, explaining the closeness of the red lines. We can observe that these neurons spike for a smaller range of speeds (Sf_n_) compared to the randomly drawn distribution (SfRand_n_).

Using an analysis similar to direction selectivity, we evaluated Sf_n_: the speed distribution for which each filter *n* spikes. However, this is not sufficient to evaluate the speed selectivity as there is a broad range of different speeds of the ball for all trajectories, but given the limited set of recorded trajectories, directions and speeds are correlated (kinematics in a uniform gravitational field), as shown in Figure 5A.

To better characterize speed selectivity, we evaluated it independently of its direction selectivity. We compared Sf_n_ with a randomly drawn distribution SfRand_n_ of ball's speeds with directions similar to the filter's direction selectivity θ*f*_n_. Details about this randomly drawn distribution and filters' speed selectivity can be found in Annex 3 in Supplementary Material. A non-selective filter should have an Sf_n_ very close to SfRand_n_. Figure 5B, shows that filters become selective to the ball's speed. These three filters with similar direction selectivity spike for a smaller range of speeds (Sf_n_) than the randomly drawn distribution (SfRand_n_), and the distributions also have different peak values. These results highlight filters' selectivity for a range of speeds and confirms previous results (Paredes-Valles et al., 2019). This selectivity is broader than direction selectivity, and although we cannot determine speed with high precision, it still provides information about velocity, which is useful to make predictions about the trajectory of the ball.

### Trajectory Prediction

After training of the SNN and PRs, the test set of trajectories was presented. For each spike generated by the last layer, the prediction was updated. We analyzed the mean Absolute Error (AE) and variability of AE (SD AE) obtained during the test phase, through the mean of separate ANOVAs, with Visibility (15–90%) as a within factor.

**Figure 6:**
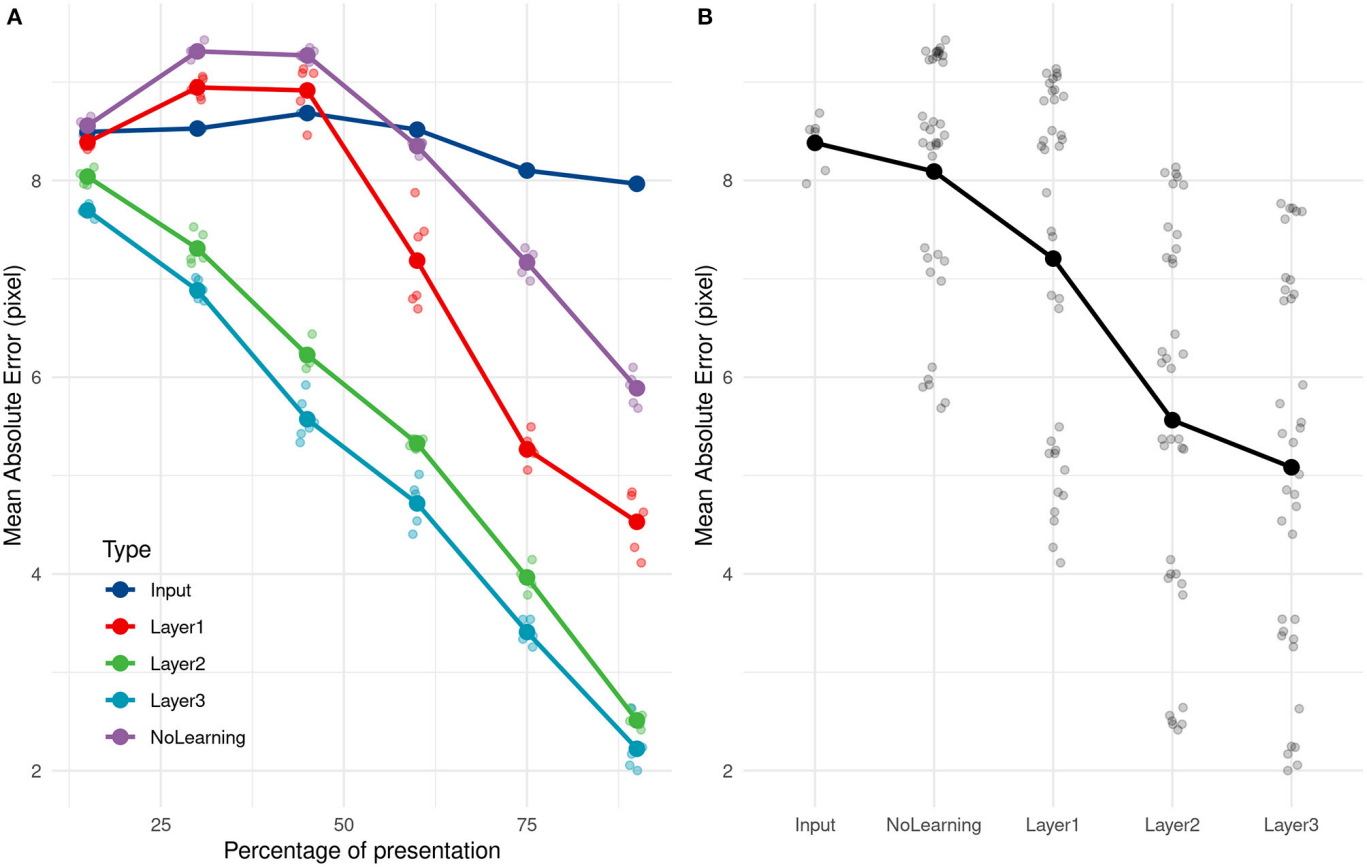
**(A)** Evolution of the average absolute error with time of presentation for different PR inputs. **(B)** Average absolute prediction error comparison for different PR inputs.

The ANOVA on AE showed that Visibility influenced the mean AE, *F*_(5, 25)_ = 1156.38, *p* < 0.001. *Post-hoc* tests using Bonferroni corrections demonstrated that the mean AE was always reduced as the visibility increased (see the layer 3 in Figure 6). We obtained good performances even with 15% of the trajectory with an error of 7.7 pixels. This error decreased with presentation time and went down to 2.2 pixels for 90% of trajectory presentation. No direction (rightward/leftward throw) error was made, whatever the percentage of presentation. It is important to note that for the lower Visibility condition (15%), the video is stopped on average 0,114 s after the throw's initiation.

As a comparison, if we compute the mean of all training values, equals to 68.2 pixel, and use it as a naive predictor, we get an error of 9.4 pixels. Even with 15% of the trajectory presentation, the SNN can predict the throw's direction and is 16.7% better than this ≪ naive ≫ predictor. The ball was still in the thrower's hand for all trajectories at 15% of trajectory presentation and also at 30% for 35 over 89 trajectories (the ball had just left the hand for others).

The analysis of SD AE also showed a significant influence of the Visibility, [*F*_(5, 25)_ = 143.60, *p* < 0.001], with the SD AE decreasing as the visibility increases. The SD goes from 5.605 pixels at 15% to 1.996 at 90%, with no significant difference between the 15 and 30% conditions, and 75 and 90% conditions. The evolution of the SD AE depending on visibility can be seen in **Figure 10B**.

We compared the prediction error over different layers, with an untrained network and with direct inputs to evaluate the performance of the learning and the impact of adding layers to the network. As shown in Figure 6, using the direct input (i.e., output of the camera), we cannot predict the reception point. As expected, PRs are not accurate with just the position information, and need more complex features, such as speed and direction encoded in filters.

Error prediction decreased over the successive layers. The first layer still made some errors but performed far better on the untrained network, more specifically for the end of the trajectory.

**Figure 7:**
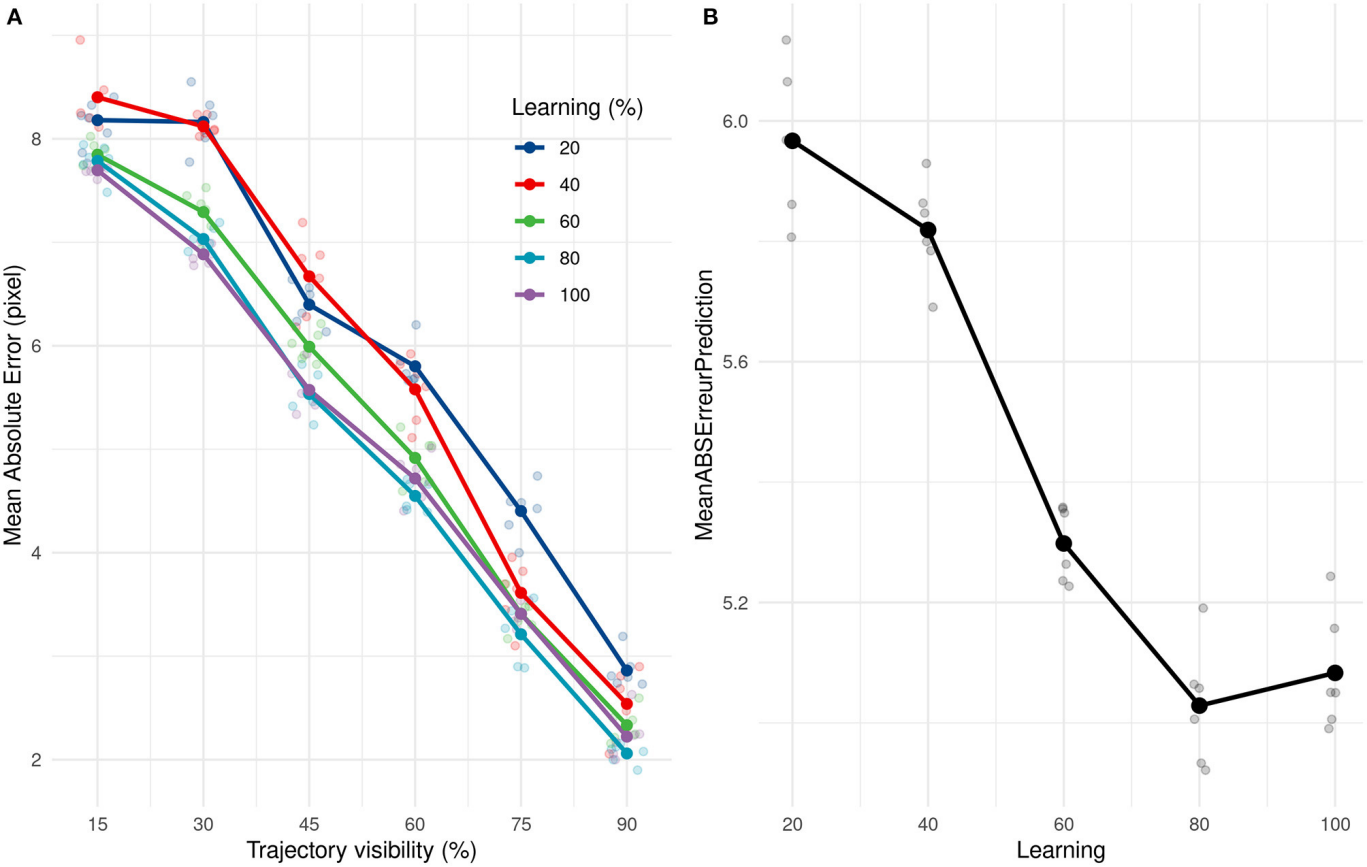
**(A)** Evolution of the average absolute prediction error with time of presentation for different number of trajectories presented during the learning phase. **(B)** Average absolute prediction error for different number of trajectories presented during the learning phase.

We subsequently investigated how quickly our solution (SNN + PR) could learn and how many presentations it needed to provide correct estimations. We applied the same learning and prediction process as before, but the learning was done with only a subset (20, 40, 60, 80, and 100%) of the 211 training trajectories. Our approach led to very good performance even when only learning from 20% of trajectories. Performances increased and then reached a ceiling at 80%, ~168 trajectories, as shown in Figure 7.

### Unsupervised Motion Tracking

**Figure 8:**
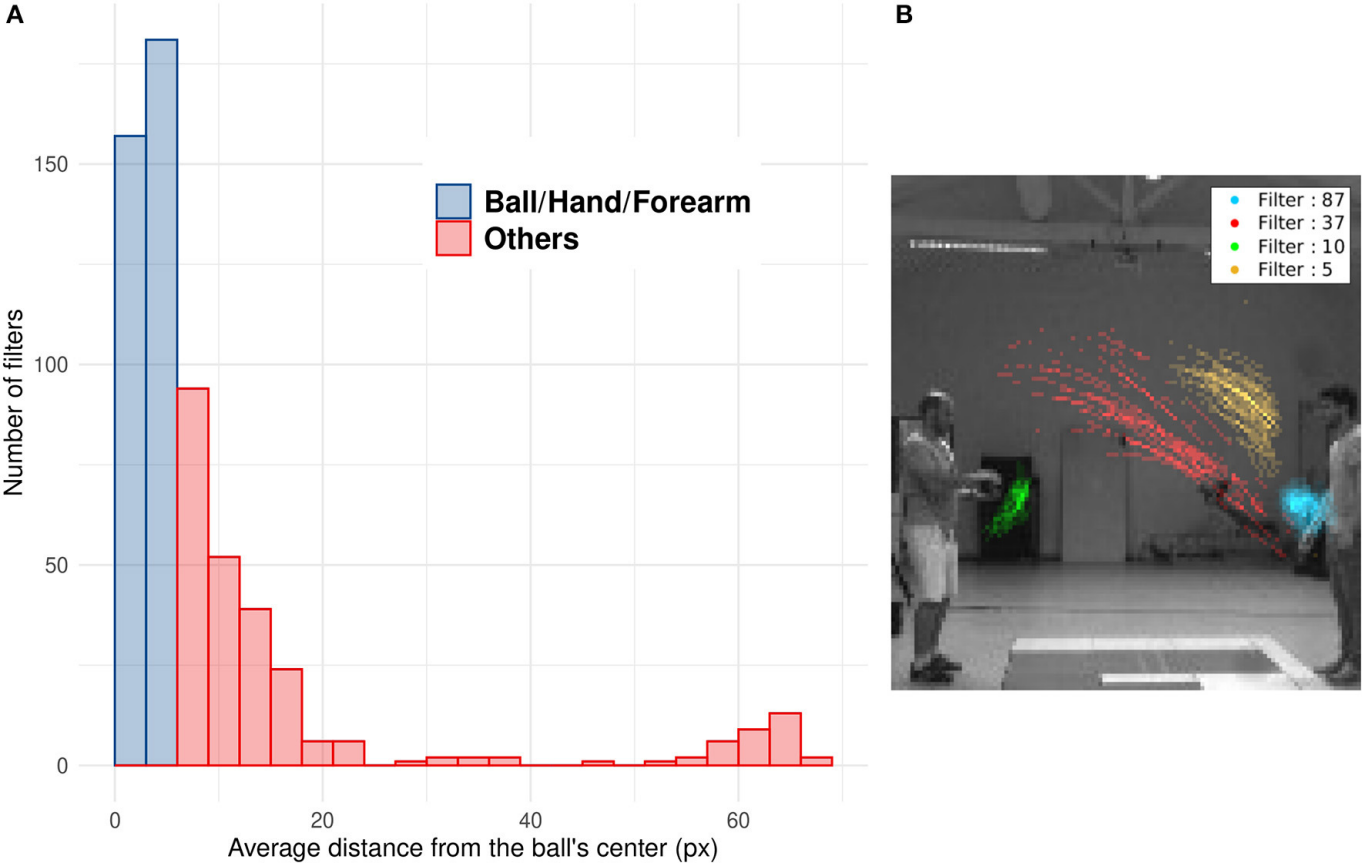
**(A)** Histogram of the mean distance Dn of all filters. Blue bars represent filters with Dn under 6 pixel which mostly spike for the ball, the hand or the forearm. Red bars represent filters which spike for other features less correlated with the ball motion (arm, after throw motion, etc.). **(B)** Spike's position for four different filters: The filter number 37 (in red) spikes for ascending leftward ball's motion, the opposite of filter 5 (in yellow) which spikes for descending rightward ball's motion. Filter 87 (in blue) is selective to the right thrower's arm during the throwing motion (i.e., when the arm rises) and filter 10 (in green) to the left thrower's arm after throwing motion (i.e., when the arm goes back to initial position).

Numerous studies showed that SNNs equipped with STDP develop a progressive selectivity to shape along their hierarchy (as more conventional neural networks) (Masquelier and Thorpe, 2007; Kheradpisheh et al., 2018; Thiele et al., 2018). Neurons in the first layers are selective to edges whereas neurons in deeper layers are selective to more complex features. In the context of our SNNs, filters with a mean distance from the ball under 6 pixels mostly encode for features related to the ball (including the forearm of the thrower when the ball is still in their hands). Filters with an higher value encode for different features, like other parts of the arm, the receiver, etc. The distribution of D_n_ highlights the ability of filters to encode for specific motions' patterns, such as the motion of the ball (see Figure 8A). As shown in Figure 8B, we can see the position of spikes for 4 different filters. Each filter spikes for specific positions and different motion patterns. This unsupervised selectivity could then be used to track motion of specific objects such as the ball, the thrower's hand, etc.

### Human's Performances

**Figure 9:**
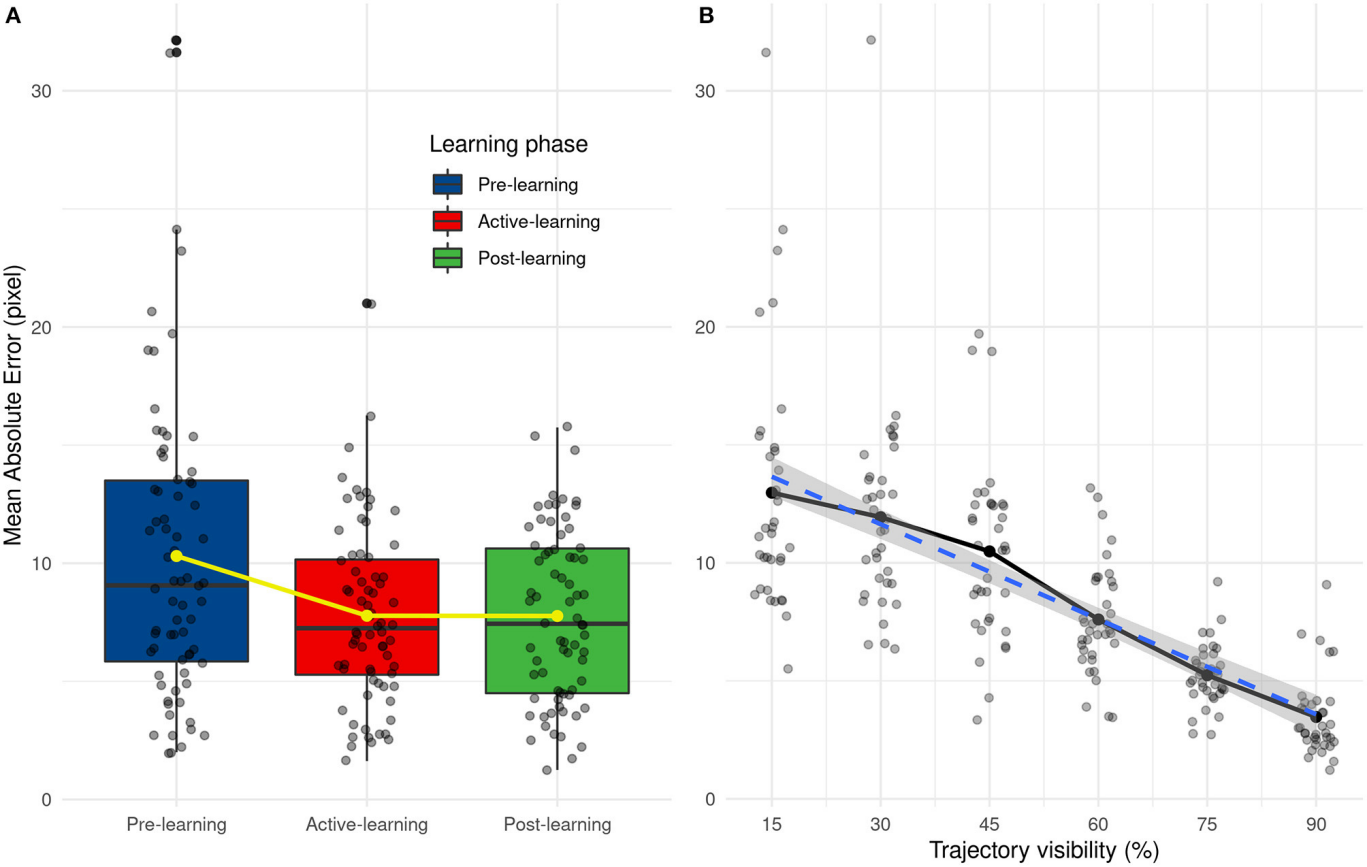
**(A)** Distribution of mean absolute error as a function of the learning phase. Yellow line and points represent average values. **(B)** Evolution of the mean absolute error depending on the trajectory visibility.

We analyzed the performance of human participants (AE and SD AE) with two separate ANOVAs, with Learning (Pre-test, Active and Post-test) and Visibility (from 15 to 90%) as within-subject variables. The results show that visibility [*F*_(5, 55)_ = 31.85, *p* < 0.001] and learning [*F*_(2, 22)_ = 10.78, *p* < 0.001] have an influence on prediction error and there are no interactions between these two factors [*F*_(10, 110)_ = 2.32, *p* = 0.016]. Prediction error decreases with the percentage of trajectory that is shown to the participants, as shown in Figure 9B. From *post-hoc* tests, we did not observe significant differences between 15 and 45 and between 45 and 60 percent of trajectory's presentation. There are also significant differences between pre-learning and other learning phases (learning and post-learning) and not between learning and post-learning, as shown in Figure 9A. These results highlight the effect of the learning phase which improves the prediction results which remain stable during the post-learning phase.

### Performance Comparison

To compare the performances between the human participants and our SNN, we performed an ANOVA on the mean AE and SD AE for the trained condition only (i.e., Post-Test condition for the humans and test set for the 6 simulations of the SNN). We used the participant-type (humans vs. SNN) as a between variable, and Visibility as a within variable.

By comparing the results between humans and SNN, a large difference between them can easily be seen at first when comparing their mean absolute error [*F*_(1, 16)_ = 47.09, *p* < 0.001] and their SD AE [*F*_(1, 16)_ = 20.16, *p* < 0.001] for each participant/simulation. This suggests that predictions made by the SNN are more accurate and more precise than human predictions.

**Figure 10:**
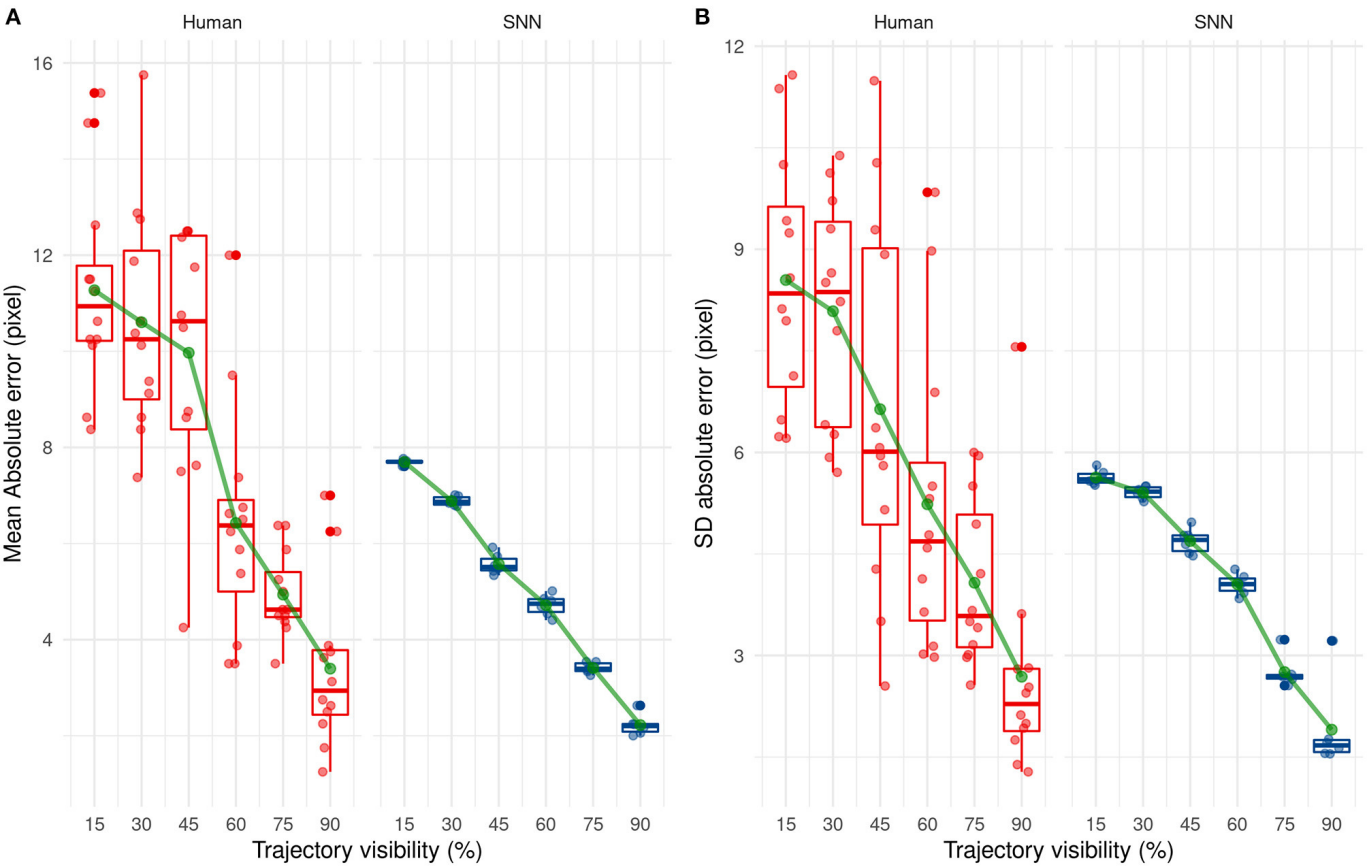
Evolution of the mean **(A)** and standard deviation **(B)** of the absolute error with trajectory visibility for humans (red) and SNNs (blue).

The mean and SD AE decreases in a linear way as Visibility increases [for the mean: *F*_(5, 80)_ = 38.21, *p* < 0.001, for the SD: *F*_(5, 80)_ = 22.88, *p* < 0.001], as shown in Figure 10. Finally, there was a significant interaction, [*F*_(5, 80)_ = 2.53, *p* = 0.036] for AE only, indicating that the SNN always outperformed human participants, except when the trajectory was presented for 60 or 90% of the trajectory. The absence of the interaction regarding SD AE [*F*_(5, 80)_ = 1.19, *p* = 0.32] indicates that SD AE is generally lower for the SNN, independent of the visibility.

**Figure 11:**
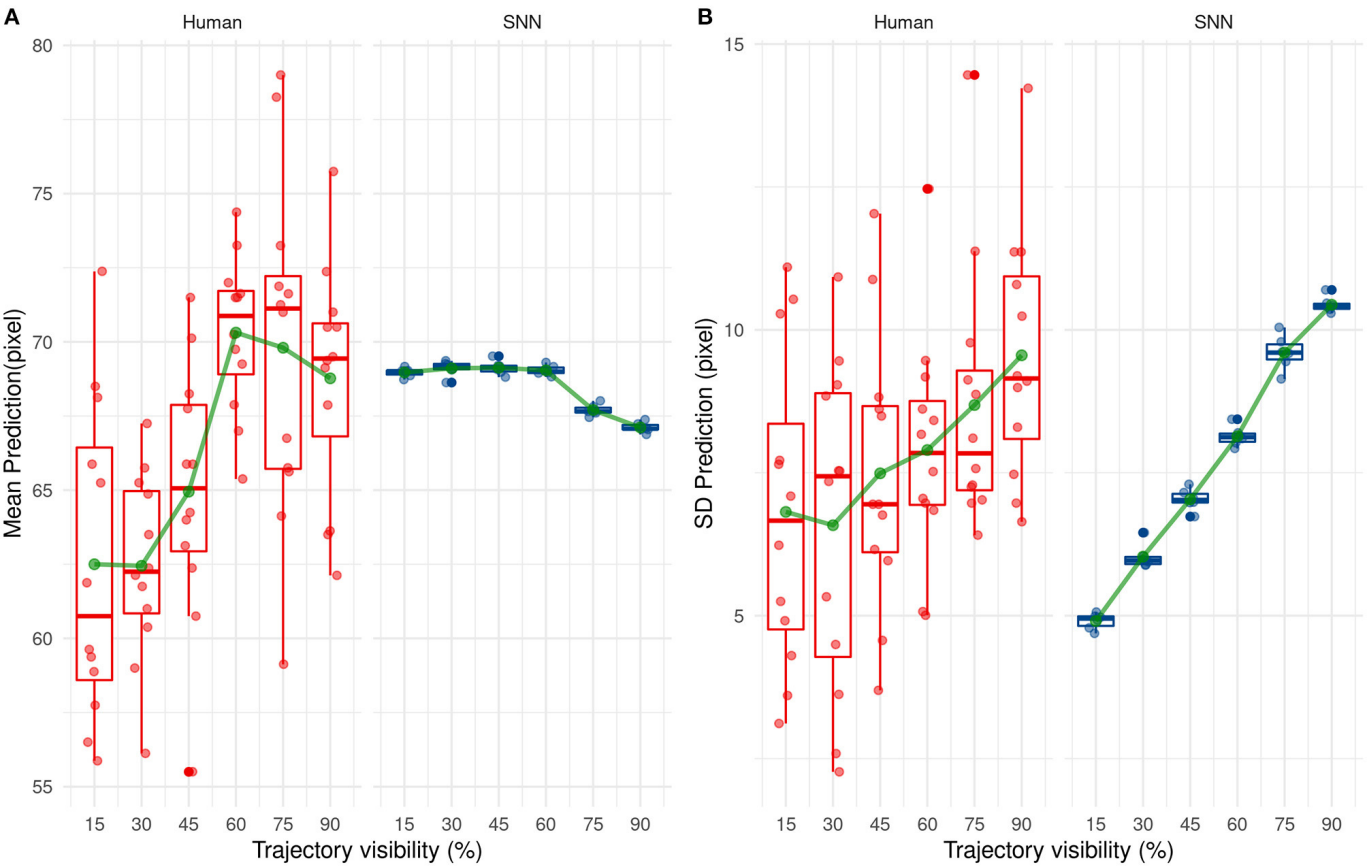
Evolution of the mean **(A)** and standard deviation **(B)** of the prediction with trajectory visibility for humans (red) and SNNs (blue).

Next, we investigated how the SNN or human participants predict the ending point of the ball trajectory. The prediction strategy used by humans and the SNN is different as shown in Figure 11. Indeed, the SNN has mean predictions close to 68 pixels which is close to the average prediction value of the training set (equals to 68.2 pixels) and remains stable over time. On the contrary, average prediction for human participants varies over presentation time. On average, human participants under-estimate the final position of the ball (prediction under 68.2 pixels) for the beginning of the trajectory, which changes to a light over-estimation with time.

The variability of predicted values is also different between humans and our system. Indeed, the standard deviation of predicted values increases over presentation's times for our solution. This one is low for the beginning of the trajectory and so our solution predicts values close to 68 px. Then, it increases the prediction's variability with more information and so presentation's time. This tendency is also valid for humans but less ≪ significant ≫ than our system.

These results show a different way to predict the reception point between our solution and humans. On the one hand, our solution makes cautious and ≪ statistical ≫ predictions by targeting close on average to 68.2 px (the average value of the training set). As visibility increases, the SNN is able to step aside from this mean value and predict a different position, close to the correct value. In other words, the SNN makes cautious predictions, based on the average value of the dataset when the visibility is low, but is able to make more liberal—but accurate—predictions as visibility increases. Humans however seem to act differently, without using a statistical mean as a target. Their perception is variable even under low visibility conditions, indicating a trial-by-trial decision, and seems to switch from an underestimation to a small overestimation as visibility increases.

## Discussion

Studies have already shown the ability of SNNs to process motion from a spike-based visual flow. This study extends the evidence of the reliability of using SNNs for motion processing and the efficiency of such networks.

One of our study's main contributions is the new sensor used to generate spikes and to analyze motion. Usually, SNNs are fed by asynchronous spiking cameras (Bichler et al., 2012; Orchard et al., 2013; Paredes-Valles et al., 2019) which spike for each step of brightness, thus generating a large number of spikes.

In this study, the brightness variation is encoded in the spike's temporality using the neuroSoc and an intensity to latency conversion rule. The filtering process used by the Neurosoc sensor thus provides less noisy and sparser information.

Firstly, we show our system is able to process motion information from a spiking camera, which is reliable for making ball trajectory predictions.

Secondly, we show that the selectivity of the filters can then be used to track specific motion patterns (arm, ball, etc.). Like the motion selectivity of our network, the tracking ability is fully unsupervised. While it was not the main objective of this study, it highlights the ability of unsupervised neural networks to solve multiple tasks. Thus, we can expect it to be relevant to other related visual tasks such as gesture recognition, counting tasks, object recognition, etc.

Finally, we show our system outperforms human prediction on this task.

Previous studies have shown that it is possible to make predictions from the outputs of SNNs receiving spikes generated by an event-based camera. Some of them use liquid state machines (LSM), but their predictions remain restricted to short durations (i.e., typically a few ms) (Burgsteiner et al., 2007; Kaiser et al., 2017). Another study used delays in anisotropic lateral connections (Kaplan et al., 2013). Others use a learning rule to anticipate inputs on longer prediction times but with simpler and repetitive input stimuli (Gibson et al., 2014). Our system permits predictions on longer duration by extracting motion features, as we used ball's trajectories restricted by physics' laws.

Event-based sensors allow performing sparse coding on dynamic visual scenes. In the context of this study, only a small part of the visual scene is relevant to be extracted as we only want to process motion (ball and arm). Using the testing dataset presented as the SNN's input, an average of about 9,000 spikes per second were generated by the neuroSoc. This represents only 0.26 percent of pixels for each frame (37 +/− 15.5 ON/OFF spikes per frame), and shows that, in comparison to a full-frame synchronous camera, the output of the NeuroSoC was extremely sparse. This, in turn, makes the system highly energy-efficient if embedded in a neuromorphic chip as the power consumption of spiking neural networks is determined by the number of spikes processed (Farabet et al., 2012). Through STDP, the network can then learn from these repeated sparse spatio-temporal stimuli and is thus highly capable of processing motion.

Even though our approach has been evaluated on a rather easy task, with little variations in trajectories and minimal background motion, this evaluation is still relevant to numerous situations such as basketball free throws, or objects moving along a specific constraint (e.g., cars moving on a road or pedestrian crossing a sidewalk). In a more complex situation with background motion, the motion tracking ability of the SNN could be used to discriminate the ball's motion from other objects.

The next step would be to evaluate our system on more complex trajectories (rebounds, collisions etc.) or scenes which involve unconstrained motion trajectories such as pedestrians moving along a footpath or players moving across a football field. This type of solution could also be useful in robotics (e.g., aerial drones) where real-time, energy-efficient processing is highly desirable.

In the near future, one of our aims is to embed this SNN on a chip such as the FPGA of the neuroSoc camera, allowing us to have the acquisition sensor, the spike extraction, and the processing (SNN) in a single, low-powered, and real-time chip. Hence, this work is the first step toward showing the reliability of a simple SNN to extract relevant spatiotemporal features using the neuroSoc camera.

## Data Availability Statement

The raw data supporting the conclusions of this article will be made available by the authors, without undue reservation.

## Ethics Statement

The studies involving human participants were reviewed and approved by University of Toulouse local ethic committee. The patients/participants provided their written informed consent to participate in this study.

## Author Contributions

RB, BC, and TM designed the project. MP developed the camera. GD adapted a code developed by TC to run the simulations, analyzed the data, and wrote the first draft of the article. All the authors provided comments on this manuscript.

## Conflict of Interest

MP is Chief Technology Officer at Yumain, which develops and commercializes the Neurosoc. Yumain has however nothing to do with the design of our study or the interpretation of the results. The SNN is currently under a patent examination process, with all the co-authors at the exception of MP listed as the inventors of this patent. The remaining authors declare that the research was conducted in the absence of any commercial or financial relationships that could be construed as a potential conflict of interest.

## References

[B1] Abderrahmane N. Miramond B. (2019). Information coding and hardware architecture of spiking neural networks, in 2019 22nd Euromicro Conference on Digital System Design (DSD) (Kallithea), 291–298. 10.1109/DSD.2019.00050

[B2] Adams S. V. Harris C. M. (2014). A proto-architecture for innate directionally selective visual maps. PLoS ONE. 9:e102908. 10.1371/journal.pone.0102908 25054209 PMC4108382

[B3] Admin (2020). Akida Neural Processor IP. BrainChip. Disponible sur: https://brainchipinc.com/akida-neural-processor-ip/ (consulté le avr. 23, 2021).

[B4] Aglioti S. M. Cesari P. Romani M. Urgesi C. (2008). Action anticipation and motor resonance in elite basketball players. Nat. Neurosci. 11, 1109–1116. 10.1038/nn.2182 19160510

[B5] Barrios-Avilés J. Iakymchuk T. Samaniego J. Medus L. D. Rosado-Muñoz A. (2018). Movement detection with event-based cameras: comparison with frame-based cameras in robot object tracking using powerlink communication. Electronics 7:304. 10.3390/electronics7110304

[B6] Bi G. Poo M. (2001). Synaptic modification by correlated activity: Hebb's postulate revisited. Annu. Rev. Neurosci. 24, 139–166. 10.1146/annurev.neuro.24.1.139 11283308

[B7] Bichler O. Querlioz D. Thorpe S. J. Bourgoin J. P. Gamrat C. (2012). Extraction of temporally correlated features from dynamic vision sensors with spike-timing-dependent plasticity. Neural Netw. 32, 339–348. 10.1016/j.neunet.2012.02.022 22386501

[B8] Brandli C. Berner R. Yang M. Liu S. C. Delbruck T. (2014). A 240 × 180 130 dB 3 μs latency global shutter spatiotemporal vision sensor. IEEE J. Solid State Circ. 49, 2333–2341. 10.1109/JSSC.2014.2342715

[B9] Burgsteiner H. Kröll M. Leopold A. Steinbauer G. (2007). Movement prediction from real-world images using a liquid state machine. Appl. Intell. 26, 99–109. 10.1007/s10489-006-0007-1

[B10] Caiman Camera. (2021). Yumain. Disponible sur: https://yumain.fr/en/products/caiman-camera/ (consulté le avr. 22, 2021).

[B11] Caporale N. Dan Y. (2008). Spike timing–dependent plasticity: a hebbian learning rule. Annu. Rev. Neurosci. 31, 25–46. 10.1146/annurev.neuro.31.060407.125639 18275283

[B12] Chauhan T. Masquelier T. Montlibert A. Cottereau B. R. (2018). Emergence of binocular disparity selectivity through hebbian learning. J. Neurosci. 38, 9563–9578. 10.1523/JNEUROSCI.1259-18.2018 30242050 PMC6705998

[B13] Delorme A. Perrinet L. Thorpe S. J. (2001). Networks of integrate-and-fire neurons using Rank Order Coding B: Spike timing dependent plasticity and emergence of orientation selectivity. Neurocomputing 38–40, 539–545. 10.1016/S0925-2312(01)00403-9

[B14] Diehl P. U. Cook M. (2015). Unsupervised learning of digit recognition using spike-timing-dependent plasticity. Front. Comput. Neurosci. 9:99. 10.3389/fncom.2015.00099 26941637 PMC4522567

[B15] Eurich C. W. Pawelzik K. Ernst U. Thiel A. Cowan J. D. Milton J. G. (2000). Delay adaptation in the nervous system. Neurocomputing 32–33, 741–748. 10.1016/S0925-2312(00)00239-3

[B16] Farabet C. Paz R. Pérez-Carrasco J. Zamarreño-Ramos C. Linares-Barranco A. LeCun Y.. (2012). Comparison between frame-constrained fix-pixel-value and frame-free spiking-dynamic-pixel convnets for visual processing. Front. Neurosci. 6:32. 10.3389/fnins.2012.00032 22518097 PMC3324817

[B17] Farrow D. Abernethy B. (2003). Do expertise and the degree of perception-action coupling affect natural anticipatory performance? Perception 32, 1127–1139. 10.1068/p3323 14651325

[B18] Faul F. Erdfelder E. Lang A. G. Buchner A. (2007). G^*^Power 3: a flexible statistical power analysis program for the social, behavioral, and biomedical sciences. Behav. Res. Methods 39, 175–191. 10.3758/BF03193146 17695343

[B19] Gibson T. A. Henderson J. A. Wiles J. (2014). Predicting temporal sequences using an event-based spiking neural network incorporating learnable delays, in 2014 International Joint Conference on Neural Networks (IJCNN) (Beijing), 3213–3220. 10.1109/IJCNN.2014.6889850

[B20] Iyer L. R. Chua Y. Li H. (2021). Is neuromorphic MNIST neuromorphic? Analyzing the discriminative power of neuromorphic datasets in the time domain. Front. Neurosci. 15:608567. 10.3389/fnins.2021.608567 33841072 PMC8027306

[B21] Kaiser J. Stal R. Subramoney A. Roennau A. Dillmann R. (2017). Scaling up liquid state machines to predict over address events from dynamic vision sensors. Bioinspir. Biomim. 12:055001. 10.1088/1748-3190/aa7663 28569669

[B22] Kaplan B. A. Lansner A. Masson G. S. Perrinet L. U. (2013). Anisotropic connectivity implements motion-based prediction in a spiking neural network. Front. Comput. Neurosci. 7:112. 10.3389/fncom.2013.00112 24062680 PMC3775506

[B23] Kheradpisheh S. R. Ganjtabesh M. Thorpe S. J. Masquelier T. (2018). STDP-based spiking deep convolutional neural networks for object recognition. Neural Netw. 99, 56–67. 10.1016/j.neunet.2017.12.005 29328958

[B24] Lee C. Panda P. Srinivasan G. Roy K. (2018). Training deep spiking convolutional neural networks with STDP-based unsupervised pre-training followed by supervised fine-tuning. Front. Neurosci. 12:435. 10.3389/fnins.2018.00435 30123103 PMC6085488

[B25] Lee J. H. Delbruck T. Pfeiffer M. (2016). Training deep spiking neural networks using backpropagation. Front. Neurosci. 10:508. 10.3389/fnins.2016.00508 27877107 PMC5099523

[B26] Lichtsteiner P. Posch C. Delbruck T. (2008). A 128$ ||times $128 120 dB 15μs latency asynchronous temporal contrast vision sensor. IEEE J. Solid State Circ. 43, 566–576. 10.1109/JSSC.2007.914337

[B27] Liu S. C. van Schaik A. Minch B. A. Delbruck T. (2014). Asynchronous binaural spatial audition sensor with 2$ || times||,$64$||,||times||,$4 channel output. IEEE Trans. Biomed. Circ. Syst. 8, 453–464. 10.1109/TBCAS.2013.2281834 24216772

[B28] Masquelier T. (2012). Relative spike time coding and STDP-based orientation selectivity in the early visual system in natural continuous and saccadic vision: a computational model. J. Comput. Neurosci. 32, 425–441. 10.1007/s10827-011-0361-9 21938439

[B29] Masquelier T. Guyonneau R. Thorpe S. J. (2008). Spike Timing dependent plasticity finds the start of repeating patterns in continuous spike trains. PLoS ONE. 3:e1377. 10.1371/journal.pone.0001377 18167538 PMC2147052

[B30] Masquelier T. Thorpe S. J. (2007). Unsupervised learning of visual features through spike timing dependent plasticity. PLoS Computat. Biol. 3:e31. 10.1371/journal.pcbi.0030031 17305422 PMC1797822

[B31] Merriaux P. Dupuis Y. Boutteau R. Vasseur P. Savatier X. (2017). A study of vicon system positioning performance. Sensors 17:1591. 10.3390/s17071591 28686213 PMC5551098

[B32] Mohammadi A. Asadi H. Mohamed S. Nelson K. Nahavandi S. (2017). “OpenGA, a C++ genetic algorithm library, in 2017 IEEE International Conference on Systems, Man, and Cybernetics (SMC) (Banff, AB), 2051–2056. 10.1109/SMC.2017.8122921

[B33] Moraitis T. Sebastian A. Eleftheriou E. (2020). Short-term synaptic plasticity optimally models continuous environments. arXiv 2009.06808 [cs, q-bio]. Disponible sur: http://arxiv.org/abs/2009.06808 (consulté le avr. 23, 2021).

[B34] Mozafari M. Kheradpisheh S. R. Masquelier T. Nowzari-Dalini A. Ganjtabesh M. (2018). First-spike-based visual categorization using reward-modulated STDP. IEEE Trans. Neural Netw. Learn. Syst. 29, 6178–6190. 10.1109/TNNLS.2018.2826721 29993898

[B35] Orchard G. Benosman R. Etienne-Cummings R. Thakor N. V. (2013). A spiking neural network architecture for visual motion estimation, in 2013 IEEE Biomedical Circuits and Systems Conference (BioCAS) (Rotterdam), 298–301. 10.1109/BioCAS.2013.6679698

[B36] Orchard G. Jayawant A. Cohen G. K. Thakor N. (2017). Converting static image datasets to spiking neuromorphic datasets using saccades. Front. Neurosci. 9:437. 10.3389/fnins.2015.00437 26635513 PMC4644806

[B37] Paredes-Valles F. Scheper K. Y. W. de Croon G. C. H. E. (2019). Unsupervised learning of a hierarchical spiking neural network for optical flow estimation: from events to global motion perception, IEEE Transactions on Pattern Analysis and Machine Intelligence, 42, 2051–2064. 10.1109/TPAMI.2019.2903179 30843817

[B38] Perrinet L. Samuelides M. Thorpe S. (2004). Sparse spike coding in an asynchronous feed-forward multi-layer neural network using matching pursuit. Neurocomputing 57, 125–134. 10.1016/j.neucom.2004.01.010

[B39] Pfeiffer M. Pfeil T. (2018). Deep learning with spiking neurons: opportunities and challenges. Front. Neurosci. 12:774. 10.3389/fnins.2018.00774 30410432 PMC6209684

[B40] Posch C. Matolin D. Wohlgenannt R. (2011). A QVGA 143 dB dynamic range frame-free PWM image sensor with lossless pixel-level video compression and time-domain CDS. IEEE J. Solid-State Circuits. 46, 259–275. 10.1109/JSSC.2010.2085952

[B41] Rawat W. Wang Z. (2017). Deep convolutional neural networks for image classification: a comprehensive review. Neural Comput. 29, 2352–2449. 10.1162/neco_a_00990 28599112

[B42] Rueckauer B. Lungu I. A. Hu Y. Pfeiffer M. Liu S. C. (2017). Conversion of continuous-valued deep networks to efficient event-driven networks for image classification. Front. Neurosci. 11:682. 10.3389/fnins.2017.00682 29375284 PMC5770641

[B43] Schrimpf M. Kubilius J. Hong H. Majaj N. J. Rajalingham R. Issa E. B.. (2018). Brain-score: which artificial neural network for object recognition is most brain-like?. bioRxiv 407007. 10.1101/407007

[B44] Son B. Suh Y. Kim S. Jung H. Kim J. S. Shin C.. (2017). 4.1 A 640 × 480 dynamic vision sensor with a 9μm pixel and 300Meps address-event representation, in 2017 IEEE International Solid-State Circuits Conference (ISSCC) (San Francisco, CA), 66–67. 10.1109/ISSCC.2017.7870263

[B45] SP730 Near-IR/Colorless Dichroic Block Shortpass Filter (2015). MidOpt. Disponible sur: https://midopt.com/filters/sp730/ (consulté le nov. 23, 2020).

[B46] Spike Event Sensor. (2021). Yumain. Disponible sur: https://yumain.fr/en/products/s-e-s-spike-event-sensor/ (consulté le avr. 23, 2021).

[B47] Taunyazov T. Sng W. See H. H. Lim B. Kuan J. Ansari A. F.. (2020). Event-driven visual-tactile sensing and learning for robots. Présenté Robot. Sci. Syst. 10.15607/RSS.2020.XVI.020

[B48] Tavanaei A. Maida A. S. (2017). Bio-Inspired Spiking Convolutional Neural Network using Layer-wise Sparse Coding and STDP Learning. *arXiv:1611.03000 [cs]*. Disponible sur: http://arxiv.org/abs/1611.03000 (consulté le avr. 12, 2021).

[B49] Thiele J. C. Bichler O. Dupret A. (2018). Event-based, timescale invariant unsupervised online deep learning with STDP. Front. Comput. Neurosci. 12:46. 10.3389/fncom.2018.00046 29962943 PMC6010570

[B50] Thorpe S. Delorme A. Van Rullen R. (2001). Spike-based strategies for rapid processing. Neural Netw. 14, 715–725. 10.1016/S0893-6080(01)00083-1 11665765

[B51] Tversky T. Miikkulainen R. (2002). Modeling directional selectivity using self-organizing delay-adaptation maps. Neurocomputing 44–46, 679–684. 10.1016/S0925-2312(02)00457-5

[B52] Van Rullen R. Thorpe S. J. (2001). Rate coding versus temporal order coding: what the retinal ganglion cells tell the visual cortex. Neural Comput. 13, 1255–1283. 10.1162/08997660152002852 11387046

[B53] VanRullen R. Guyonneau R. Thorpe S. J. (2005). Spike times make sense. Trends Neurosci. 28, 1–4. 10.1016/j.tins.2004.10.010 15626490

