# Supplementary material for: Event-Based Trajectory Prediction Using Spiking Neural Networks
Source: Front Comput Neurosci. 2021 May 24;15:658764. doi: 10.3389/fncom.2021.658764 (PMC8180888; doi:10.3389/fncom.2021.658764)
Supplement: Supplementary file 1 [file Data_Sheet_1.pdf]

Annex 1 – Lateral inhibition and threshold adaptation

As explained in section 2.2.4, a lateral inhibition and a threshold variation mechanism was used to prevent filters from learning similar features and increase the network's sparsity and selectivity.

A time dependent value was added to the threshold for all neurons sharing the same retinotopic position as the spiking neuron, as shown in equations 5 and 6.

$$U_{AT,n}=\sum_{k=i}^{i+N_f} U_{Long,k}(t) \quad \text{and} \quad U_{Thresh,n}(t)=U_{Thresh}+U_{AT,n}(t) \quad (5)$$

With:

- i: index of the first neuron connected to the same patch of the spiking neuron n

- N_f: The filter's number for the corresponding layer

$$U_{Long,n}=U_{MaxThresh} * (t-t_n)/T_{Thresh}\, if \quad t-t_n \leq T_{Thresh}$$

$$U_{Long,n}=U_{MaxThresh} * \left(1-\left((t-t_n)-T_{Thresh}\right)/T_{Thresh}\right) \quad if \quad t-t_n > T_{Thresh} \quad \text{and} \quad t-t_n < 2\,T_{Thresh} \quad (6)$$

$$U_{Long,n}=0 \quad otherwise$$

With:

- t: current time

- t_n: last spike of neuron n

- $U_{AT,n}$: intensity of the threshold adaptation for the neuron n

The lateral inhibition value U_{Inst} and the amplitude of the threshold adaption $U_{MaxThresh}$ are proportional to $U_{PropInh}$. Which is dependent on membrane potentials of neurons connected to same patch as the spiking neuron, as shown in equation 7. This kind of process has the advantage of generating strong inhibitions at the beginning of the learning phase. Indeed, the filters have a broad range of selectivities, and all neurons can potentially spike for a given stimulus. When the first neuron of the patch spikes, all others are close to the threshold value. The overall membrane potential of these neurons is high, leading to a

higher value of $U_{PropInh}$, preventing filters from learning similar patterns. During the learning, the filters' selectivity becomes sharper and only a few neurons are highly excited when a given stimulus is presented. Other neurons, non-selective to this stimulus, will register a low membrane potential value, generating a lower $U_{PropInh}$.

The instantaneous inhibition value and the longer threshold adaptation value $U_{MaxThresh}$ are equal to $U_{PropInh}$ multiplied by a factor f_{Inst} and f_{Long}, equation 8. All current active threshold adaptation process linked to neurons connected to the same patch are summed to generate $U_{AT,\,n}$, see equation 5.

$$U_{PropInh} = -\sqrt{\frac{1}{N_{RF}} \sum_{n=i}^{i+N_{RF}} \left(U_n(t) - U_{AT,n}(t)\right)^2} \qquad (7)$$

$$U_{MaxThresh} = f_{Long} * U_{PropInh} \quad \text{and} \quad U_{Inst} = f_{Inst} * U_{PropInh} \; (8)$$

With :

- U_n: membrane potential value of the neuron n

Annex 2 – Score mechanism

The prediction was made on two dimensions,. The first one, the X dimension was simplified with only twho choices, leftward or rightward directions. For each filter n, we defined a value $XPred_n$ between zero and one, which is the ratio of spike counts for leftward direction to all generated spikes by this filter n. A value close to zero means that the filter mostly spikes for rightward directions, and otherwise for leftward directions when the value is close to one.

As explained in section 2.4, we used polynomial regressions to predict the y-direction. A scoring mechanism was used to spatially integrate the predicted value $Y_{Pred,n}$ based on the PR's reliability, and perform an average prediction over time.

Two Sc_{Pred} vectors with a length equal to 120 (height of the frame and one for each side) were defined and contained scores, one for each direction. Predictions made at the end of the trajectory were more reliable than previous predictions. Indeed, it is harder to make predictions at the beginning of the trajectory, when the ball is still in the thrower’s hand than a few milliseconds before the receiving point. To give more impact to the latest predictions, we add a decay to the score vector Sc_{Pred}, as shown in equation 9. Scores were updated for each spike. A prediction was made, depending on the filter and position

of the neuron. A maximum value was added to the prediction made by the current PR. A decreasing value was also added to some values depending on the RMSE of the PR as shown in the figure 3, panel A. We ensured that the cumulated value was always equal to 1 (area of each line blue, red and yellow are equals to 1). These values were then divided by the mean distance to the ball of the filter D_n, to give more weights to filters encoding for ball motion than others encoding for the receiver's arm for example.

These values were added to the predicted direction vector, which means the rightward vector if X_{pred_n} is under 0.5 or the leftward vector otherwise.

The index of the maximal value was finally selected as the predicted value.

$$Sc_{Pred} = Sc_{Pred} \cdot \exp\left(\frac{-\Delta t}{\tau}\right) \quad (9)$$

Annex 3 – Speed selectivity

As mentioned in section 3.1.2, to evaluate filters' speed selectivity, we compared Sf_n the speed distribution for which each filter n spikes, with Sf_{Rand_n}, a randomly drawn speed distribution based on filter selectivity θf_n.

For each direction, we selected a number of speed values proportional to the number of spikes for this direction by a filter n. These values were drawn randomly from all ball velocities with a similar direction. It gave us a distribution of speeds correlated to the filter direction's selectivity, as illustrated in the figure X below.

For each filter n, we also evaluated the ratio between ω_n and ω_{rand_n}, the standard deviation of Sf_n and Sf_{Rand_n} respectively. We then calculated a speed selectivity score Ω_n, the ratio between ω_n and ω_{rand_n} (equation 10). We evaluated Ω_{All}, the average Ω_n for all neurons, weighted by the amount of spikes by filter (equation 10). We obtained an Ω_{All} of 0.38, significantly under a value of 1. The ω_n value is thus lower than ω_{rand_n}, highlighting a selectivity for speed.

$$\Omega_n = \frac{\omega_n}{\omega rand_n} \quad \text{and} \quad \Omega_{All} = \frac{\sum \Omega_n \cdot C_n}{\sum C_n} \quad (10)$$

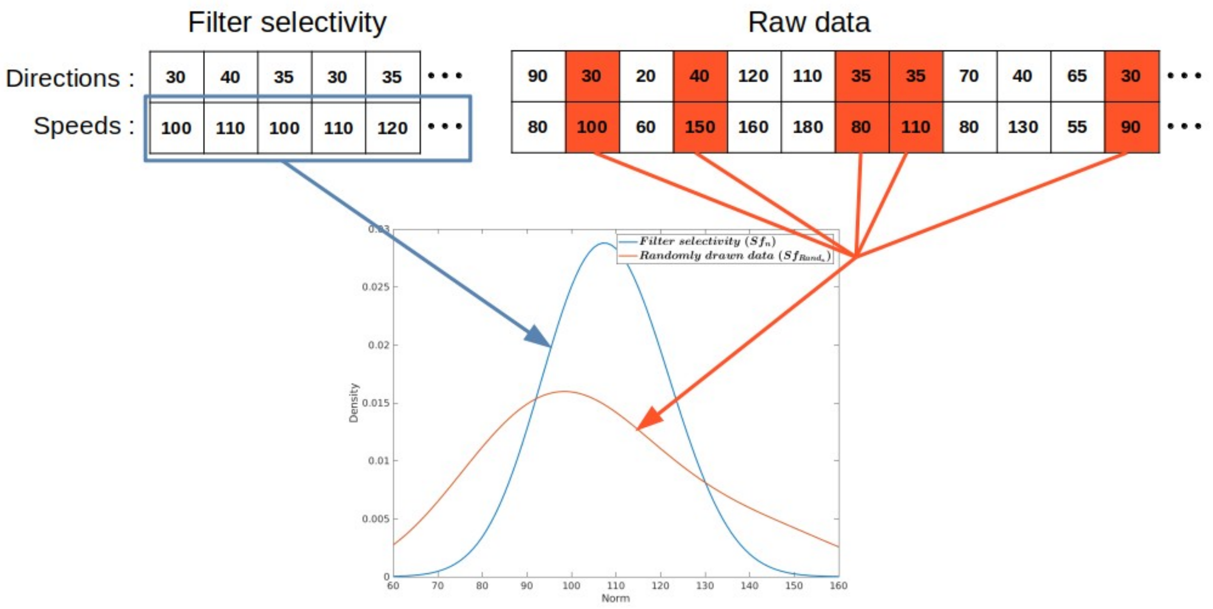

Figure 12: *Selection process of random speeds based on filter's direction selectivity. Speeds with directions similar to the filter direction selectivity are drawn randomly from all generated speeds and directions to generate* Sf_{rand_n}
